# Scrub Typhus, Republic of Palau

**DOI:** 10.3201/eid1202.050967

**Published:** 2006-02

**Authors:** Linda J. Demma, Jennifer H. McQuiston, William L. Nicholson, Staci M. Murphy, Pearl Marumoto, J. Maireng Sengebau-Kingzio, Stevenson Kuartei, A. Mark Durand, David L. Swerdlow

**Affiliations:** * Centers for Disease Control and Prevention, Atlanta, Georgia, USA;; † Ministry of Health, Koror, Republic of Palau;; ‡ Department of Health Services, Colonia, Federated States of Micronesia

**Keywords:** Orientia tsutsugamushi, scrub typhus, rickettsial disease, zoonotic disease, Tsutsugamushi disease, vector-borne disease, research

## Abstract

Scrub typhus, caused by *Orientia tsutsugamushi*, is a severe febrile illness transmitted to humans by trombiculid mites, which normally feed on rodents. The first known outbreak of scrub typhus in Palau occurred in 2001 to 2003 among residents of the remote southwest islands. To determine the extent of scrub typhus distribution in Palau, we tested serum samples from humans and rodents for antibodies to *O. tsutsugamushi*. Of 212 Palau residents surveyed in 2003, 101 (47.6%) had immunoglobulin G (IgG) antibody titers >1:64, and 56 (26.4%) had concurrent IgG and IgM antibody titers >1:512 and 1:64, respectively. Of 635 banked serum samples collected from Palau residents in 1995, 34 (5.4%) had IgG antibody titers >1:64. Sera collected from rodents (*Rattus norvegicus* and *R. rattus*) in 2003 and 2005 were tested, and 18 (28.6%) of 63 had IgG antibody titers >1:64. These findings suggest that scrub typhus is endemic in Palau.

## Abstract 2

Scrub typhus is likely endemic in Palau.

Scrub typhus is a zoonotic illness caused by *Orientia tsutsugamushi*. The pathogen is transmitted through the bite of larval mites (chiggers) of the *Trombiculidae* family, which serve as both the vector and the reservoir (*1**,**2*). Rodents of the family *Muridae* (rats and mice) are common hosts for trombiculid mites and may support *O. tsutsugamushi*. Geographically specific foci of scrub typhus are thus determined by the distribution of vector mites and their rodent hosts and by interactions of mites and rodents with humans (*3*). Scrub typhus has been reported from many regions of Asia and the Pacific islands, and known disease-endemic regions extend from Japan and eastern Russia southward to Australia and westward to Pakistan and Afghanistan (*4**,**5*).

Scrub typhus is typically a nonspecific febrile illness; its severity may be influenced by the strain of *O. tsutsugamushi*, a person's immune status, and other factors. Diagnosis may be complicated in areas where the disease has not been documented recently or in regions lacking the capacity for laboratory confirmation. Illness develops after an incubation period of 6 to 21 days and usually begins with an eschar at the site of a chigger bite. Fever, headache, and myalgias are common, and a maculopapular rash may also be present. Nausea, vomiting, diarrhea, or lower respiratory symptoms can also occur. Manifestations such as pneumonitis, meningoencephalitis, jaundice, renal failure, and myocarditis can develop during the prolonged clinical course of untreated illness (*6*). Establishing the diagnosis and initiating prompt antimicrobial drug therapy are important because death rates for untreated scrub typhus patients are 1%–30% (*5*). Scrub typhus is effectively treated with doxycycline, and treatment should begin immediately upon suspicion of illness without awaiting laboratory confirmation.

From October 2001 to October 2003, an outbreak of scrub typhus was confirmed among residents of the Republic of Palau, a Pacific island nation 900 km east of the Philippines (Figure 1). The outbreak occurred among residents of several remote southwest islands (*7*). These islands, ≈300 km from the capital of Koror, are difficult to reach, and affected persons required emergency evacuation by boat to Koror for treatment. This outbreak affected primarily children, and illness was characterized by fever and severe abdominal distress (*7*). Infection with *O. tsutsugamushi* was confirmed by serologic testing at the Centers for Disease Control and Prevention (CDC), where extremely high titers of antibodies to *O. tsutsugamushi* were demonstrated in patient serum specimens (IgG range 1:2,048–1:262,144, IgM range 1:1,024–1:16,384) (*7*). Before this outbreak was confirmed, scrub typhus had not been recognized in Palau. To better direct efforts to control the disease, Palauan public health officials needed to determine whether *O. tsutsugamushi* was restricted to these remote southwest islands or whether the pathogen was present in other parts of Palau. In addition, public health officials wanted to ascertain whether *O. tsutsugamushi* had been recently introduced to Palau or whether it is endemic but poorly recognized. We conducted an investigation in 2003 and 2005 to assess antibodies to *O. tsutsugamushi* among humans and rodents from various regions of Palau. In addition, we assessed the historical presence of scrub typhus by examining banked serum collected from residents of Palau in 1995.

**Figure 1:**
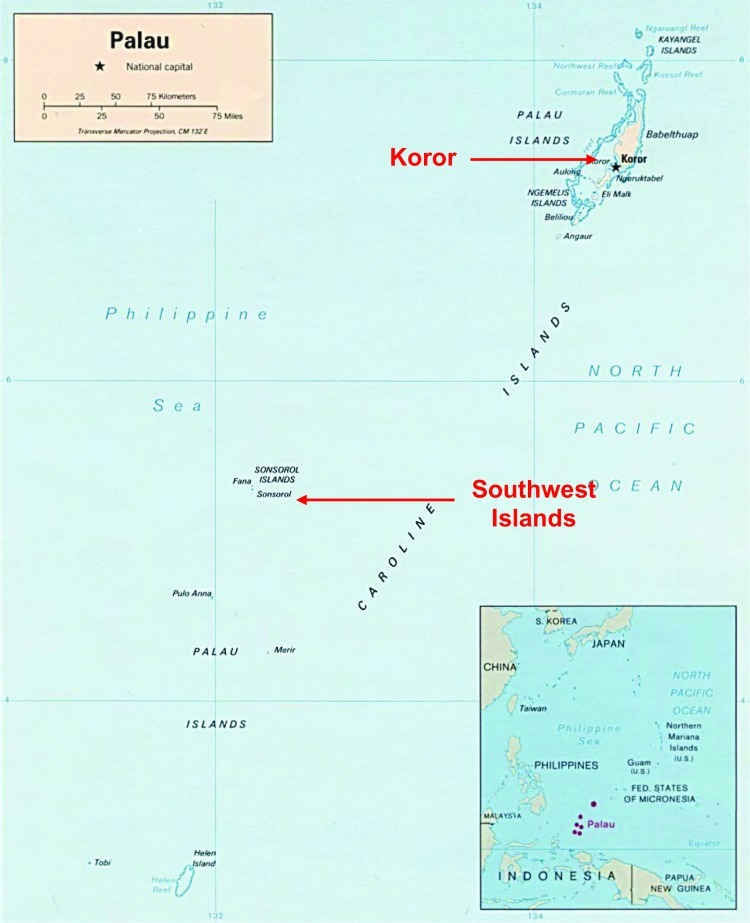
The Palau Islands. Map courtesy of the Central Intelligence Agency, 2004 (available from http://www.cia.gov/cia/publications/factbook/geos/ps.html).

## Methods

### Human Serosurvey, 2003

A prospective serologic survey was conducted among residents of Palau in December 2003. Three distinct groups were assessed: 1) residents of the southwest islands, 2) residents of Echang hamlet (a community within Koror inhabited by migratory southwest island residents and their families), and 3) residents of other Koror hamlets. Although residents frequently move between the southwest islands and Echang, they seldom migrate from these areas to other hamlets in Koror.

Serum samples from consenting residents were tested for antibodies to *O. tsutsugamushi* (Karp strain) by indirect immunofluorescence assay (IFA) and described previously (*7**,**8*). Antigen suspensions from the Karp strain of *O. tsutsugamushi* were prepared in chicken yolk sac and pipetted onto slides coated with bovine serum albumin (BSA, 1% in sterile water), air dried, fixed with acetone, and stored at –75°C until use. Slides were warmed to room temperature in desiccated conditions. Serial 2-fold dilutions, beginning at 1:16, were made in sample diluent (phosphate-buffered saline [PBS], pH 7.38, with 1% BSA and 1% normal goat serum) and added to slides for 30-min incubation at 37°C, followed by washing in PBS, pH 7.38, for 15 min (3 washes × 5 min). An optimized dilution (1:150) of fluorescein isothiocyanate (FITC)–labeled goat antihuman conjugate IgG (γ-chain-specific) (Kirkegaard & Perry Laboratories, Inc., Gaithersburg, MD, USA) was then applied to the slides, which were incubated and washed as before; Eriochrome Black T counterstain was added to the middle wash. After glycerol-PBS mounting medium and coverslip were applied, the slides were read at a magnification of 400× with an epifluorescence UV microscope. Any reactive samples were then titrated to endpoint by using IgG-specific (γ) conjugate. Titers were recorded as the reciprocal of the highest dilution displaying specific fluorescence. For IgM testing, the samples were first depleted of IgG by using a recombinant protein G device (Rapi-Sep-M kit, Pan Bio, Columbia, MD, USA). This procedure resulted in a final 1:8 dilution of the serum sample, which was then diluted further in sample diluent and placed onto slides. The protocol is similar to that detailed above for IgG, but it used FITC-labeled, goat antihuman IgM (μ-chain specific) conjugate at a working dilution of 1:100.

For specimens with an anti–*O. tsutsugamushi* IgG antibody titer >1:16, endpoint titers were determined for IgG and IgM by serial dilution of samples. An IgG antibody titer >1:64 was considered seropositive and indicated past exposure to *O. tsutsugamushi*. Concurrent IgG and IgM antibody titers >1:512 and >1:64, respectively, were considered evidence of possible recent exposure to *O. tsutsugamushi*, based on assessment of serum samples collected from southwest islands scrub typhus patients 5 months to 2 years after infection (Table 1).

**Table 1:** Results of *Orientia tsutsugamushi* IFA serologic testing of scrub typhus patients from the southwest islands of Palau, 5 months to 2 years after illness onset*

Patient	Days since illness onset	IgG antibody titer	IgM antibody titer
1	160	1:8,192	1:64
2	245	1:2,048	1:256
3	335	1:512	1:128
4	375	1:2,048	1:128
5	505	1:1,024	1:64
6	785	1:4,096	1:256

*IFA, indirect immunofluorescence assay; Ig, immunoglobulin.

Questionnaires were administered to residents who provided blood specimens for the serosurvey. We collected information on history of febrile illness and residence or travel history within the past 2 years and on recreational and occupational activities. Epidemiologic and serologic data were analyzed by using EpiInfo 2002 (*9*) and the statistical package SPSS for Windows 12.0 (standard version, SPSS Inc., Chicago, IL, USA). Geometric mean titers (GMTs) were compared between locations by the nonparametric Kruskal-Wallis and Mann-Whitney tests, accounting for multiple comparison groups. All univariate analyses were conducted to account for the cluster design of the survey, with household as the primary sample unit (*10*).

### Human Serosurvey, 1995

Serum specimens collected from residents of Palau during a 1995 dengue outbreak investigation were examined retrospectively for antibodies to *O. tsutsugamushi* (*11*). Samples had been stored frozen at –70°C since 1995. Samples were considered exempt from human subjects review after the removal of all identifying information so we could not obtain patient information or epidemiologic data. IFA was performed; IgG antibodies reactive with *O. tsutsugamushi* at a titer >1:64 indicated exposure to scrub typhus (*8*).

### Rodent Surveys

Rodent trapping and sample collection were conducted in December 2003 and April 2005. Endpoint IgG antibody titers reactive to *O. tsutsugamushi* were determined by serial dilution of samples and IFA similar to that as described above for human serum samples (*8*), using a goat anti-rat IgG (γ) conjugate and positive and negative rat serum as controls. Serum specimens with an IgG antibody titer >1:64 were considered seropositive.

In addition, a survey of rodent activity was conducted for households visited during the prospective human serosurvey. Households were scored according to the following 3 observational categories: 1) presence of actual rodent sites, including visible evidence such as footprints, holes, and droppings; 2) appearance of potential rodent sites, including visible evidence of environmental situations that might support rodents, such as piles of debris or trash, unsealed sewers, and refuse pits, and 3) reported rodent activity by household members (reports of sightings, noises, odor, or debris, such as discarded food).

## Results

### Human Serosurvey, 2003

During the investigation, 212 blood samples were collected from consenting residents of 88 households, including 22 households from the southwest islands, 29 households from Echang, and 37 households from other Koror hamlets. The median age of persons from whom blood was collected was 28 years for the southwest islands, 36 years for Echang, and 36 years for other Koror hamlets; 37 (62.7%), 23 (42.6%), and 53 (53.5%) of persons were male for the southwest island, Echang, and other Koror hamlets, respectively. The average number of persons per household was 3.2, 6.8, and 5.6 for the southwest islands, Echang, and other Koror hamlets, respectively. The proportion of the overall population sampled was ≈80% for the southwest islands, 18% for Echang, and 0.78% for other Koror hamlets.

**Figure 2:**
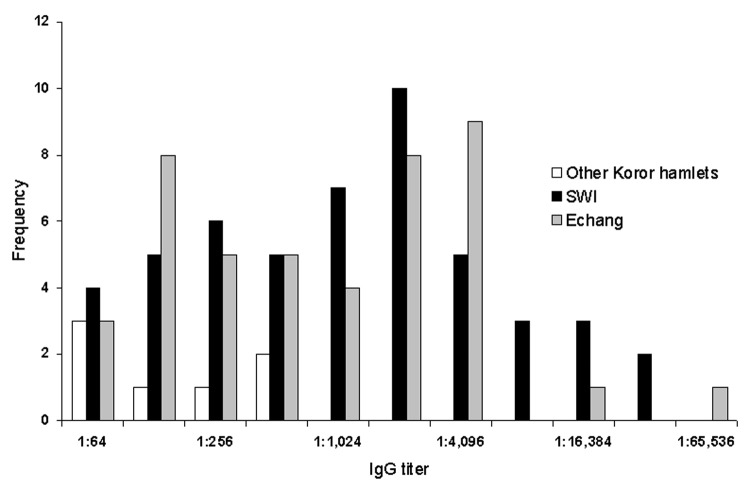
Anti–*Orientia tsutsugamushi* immunoglobulin G antibody titers by indirect immunofluorescent antibody assay for Palau residents, 2003. SWI, southwest islands.

To demonstrate the range of titers observed and the differences between locations, the frequency of IgG titers in each location is shown in Figure 2. A summary of serologic results is presented in Table 2.

**Table 2:** Results of *Orientia tsutsugamushi* IFA serologic testing of Palau residents, 2003*

	SWI, n = 59 (%)	Echang, n = 54 (%)	Other Koror hamlets, n = 99 (%)
No. IgG >1:64	50 (84.7)	44 (82)	7 (7.1)
No. IgG >1:512 and IgM >1:64	32 (54)	22 (41)	2 (2)
Geometric mean IgG titer	996	740	102
Median IgG titer (range)	1:1,024 (1:32–1:32,768)	1:1,024 (1:16–1:65,536)	1:64 (1:16–1:512)

*IFA, indirect immunofluorescence assay; SWI, southwest islands; Ig, immunoglobulin.

GMTs differed significantly among residents from different locations. Specifically, GMTs for southwest island and Echang residents were significantly higher than those for residents from other Koror hamlets (p = 0.004 and p = 0.002, respectively). Southwest island residents were significantly more likely than residents of other Koror hamlets to be seropositive (risk ratio [RR] 6.09, 95% confidence interval [CI] 3.33–11.14, p<0.001). Echang residents were also significantly more likely to be seropositive than were residents of other Koror hamlets (RR 5.02, 95% CI 2.86–8.80, p<0.001). Residents of the southwest islands and Echang did not differ significantly in seropositive status.

The median age of seropositive persons was 30 years for southwest island residents, 35 years for Echang residents, and 30 years for residents of other Koror hamlets. In the southwest islands, residents >18 years of age were significantly more likely to be seropositive than were children (RR 1.35, 95% CI 1.00–1.82). No children were seropositive in Echang, and no significant difference in past exposure between age groups in residents of other Koror hamlets was evident. Among persons with evidence of possible recent exposure (concurrent IgG >1:512 and IgM >1:64), 25 (78.1%) of 32 southwest island residents, all (100%) Echang residents, and both (100%) residents of other Koror hamlets were adults >18 years old.

Of the 56 Palau residents with evidence of possible recent exposure to scrub typhus (concurrent IgG >1:512 and IgM >1:64), 15 (26.8%) reported that they had not traveled to the southwest islands or other islands during the past 2 years. In addition, neither of the 2 residents residing within other Koror hamlets with evidence of possible recent exposure to *O. tsutsugamushi* reported visiting Echang hamlet in the past 2 years, which suggests that their exposures occurred elsewhere in Palau.

### Human Serosurvey, 1995

Serum samples collected from Palau residents during a 1995 dengue outbreak investigation were also tested for evidence of IgG antibodies to *O. tsutsugamushi*. Of 635 specimens tested, 34 (5.4%) were positive at a titer >1:64.

### Rodent Survey

A total of 63 rodents were trapped on Palau in 2003 and 2005, including 5 from the southwest islands, 23 from Echang, and 35 from other Koror hamlets. Rodents were identified primarily as *Rattus norvegicus* (Norway or brown rat), although 6 from Echang were identified as *R. rattus* (black or roof rat). All 5 rats (100%) collected in the southwest islands had IgG antibody reactive to *O. tsutsugamushi* at titers >1:64 (GMT 1:112, range 1:64–1:8,192). In addition, IgG antibodies to *O. tsutsugamushi* were detected in 4 (17.4%) of 23 rats from Echang (GMT 1:24, range 1:16–1:128) and 9 (25.7%) of 35 rats from other Koror hamlets (GMT 1:32, range 1:16–1:2,048).

A survey to assess rodent activity was conducted at households in the southwest islands, Echang, and other Koror hamlets that were visited as part of the human serosurvey. Significantly more actual and potential rodent sites were observed in the southwest islands and Echang than in other Koror hamlets (Table 3, p<0.001).

**Table 3:** Evidence of rodents around households in Palau, 2003*

Evidence	SWI	Echang	Other Koror hamlets
Average no. actual rodent sites per household	1.55†	2.34†	1.17
Average no. potential rodent sites per household	3.41†	2.55†	1.61

*SWI, southwest islands. †Significantly greater than Koror, p<0.001.

## Discussion

After *O. tsutsugamushi* was identified as the cause of an outbreak of severe illness among residents of Palau from 2001 to 2003 (*7*), officials were concerned about what was perceived to be a newly emergent disease in the remote southwest islands. This investigation was conducted to determine the historical presence and current distribution of scrub typhus among rodent reservoirs and human hosts in Palau to better direct efforts to control disease. We found widespread seroprevalence of antibodies to *O. tsutsugamushi* among both humans and rodents from several areas of Palau, including the southwest islands, Echang, and other Koror hamlets. Although the 2001–2003 outbreak involved only patients from the southwest islands, and to date no patients have been identified from Koror, our data show that scrub typhus is likely endemic in many areas of Palau. We also identified antibodies to *O. tsutsugamushi* among banked serum samples collected from residents of Palau in 1995, which suggests that the disease has been present in the region for at least a decade. Thus, the 2001–2003 outbreak of scrub typhus in the southwest islands is unlikely to be a result of a recent introduction of the pathogen and is probably related to unique host and environmental factors that increased occurrence or recognition of an established disease.

Although the 3 areas had significant differences in seroprevalence, we did not observe any significant differences in individual or household risk factors for seropositive status between these geographic areas (data not shown). We did observe differences in general household environments and individual activities between the 3 geographic areas. Specifically, residents of Echang and the southwest islands appeared to be more frequently exposed to rodents and outdoor environments where mite exposure might be expected to be increased. Because southwest island residents were younger, they may be more likely to engage in recreational activities that place them at increased risk for mite exposures. In addition, residents of Echang and the southwest islands were often fishermen or construction workers and thus more likely to engage in outdoor occupational activities.

These data are subject to several limitations. We did not evaluate the possible influence of immunologic cross-reactivity between *O. tsutsugamushi* and other disease agents; however, *O. tsutsugamushi* is antigenically distinct from other rickettsiae, and cross-reactivity is thought to be minimal. The criteria used to define a possible recent exposure to *O. tsutsugamushi* were determined through assessment of scrub typhus patients from the southwest islands who were tested 6 months to 2 years after infection; however, because the sample size used for this determination was small, we cannot predict the sensitivity of this designation. Furthermore, we cannot rule out the possibility of reexposure as a possible explanation for elevated titers in persons assessed in the serosurvey nor quantify how reexposure may influence our estimation of recent versus past exposure. Finally, the retrospective human serosurvey used specimens collected in 1995 from clinically ill patients as part of a dengue fever outbreak, and long-term storage of these specimens may have influenced detectable antibody titers. In contrast, the 2003 human serosurvey included only healthy residents, and serum samples were tested within 1 year of collection.

Although no human cases of scrub typhus have been recognized to date among residents of the main island of Koror, this investigation indicates that 41% of residents of Echang and 2% of residents of other Koror hamlets had serologic evidence that suggested a possible recent exposure to scrub typhus. The clinical manifestations of scrub typhus are often nonspecific and are similar to those of other endemic zoonotic and vectorborne diseases in Palau, such as leptospirosis and dengue fever. In addition, the severity of disease associated with scrub typhus can be highly variable; the disease may be milder among persons with partial prior immunity. No laboratory testing for scrub typhus was conducted before the 2001–2003 outbreak. Thus, cases of scrub typhus were likely occurring on the main island of Koror but were unrecognized or masked because of the presence of other, clinically similar, endemic diseases.

Eschars or rashes, which are characteristic of scrub typhus infection, may arouse clinical suspicion, but they may be difficult to observe in darker skinned persons, including Pacific islanders. In addition, eschars are less frequently reported in regions where the disease is hyperendemic because of partial immunity from prior exposures (*5*). None of the patients identified during the 2001–2003 outbreak on the southwest islands had an eschar recorded. The absence of severe disease among Palau residents with serologic evidence of recent exposure, as well as the absence of reported eschars among scrub typhus patients from the 2001–2003 outbreak, lends further support to the endemicity of scrub typhus in the region.

The location of Palau and its similarity in terrain and climate to other known disease-endemic regions suggest that this environment might readily support an endemic focus of scrub typhus. The exact role of rodents in distribution and transmission of *O. tsutsugamushi* is not well elucidated, but the detection of rats with antibodies in Palau suggests infected mites and thus indicates a risk for humans to acquire infection (*1**–**3**,**12*). Because rats are the common host for the mite that transmits *O. tsutsugamushi*, rodent burrows in close proximity to humans are a substantial and controllable risk factor. This investigation showed that households in the southwest islands and Echang were significantly more likely to have evidence of rodents than were other hamlets in Koror and might benefit from targeted rodent control programs.

The results of our investigation demonstrate the presence of *O. tsutsugamushi* throughout Palau, and historical assessments provide evidence that the disease has been present in the region as early as 1995. Although human cases of scrub typhus appear to be currently limited to the remote southwest islands of Palau, the serologic evidence of exposure to *O. tsutsugamushi* in Echang and other hamlets of Koror indicates that outbreaks could emerge in these locations. Active surveillance for human cases, coupled with appropriate laboratory diagnostics, has been implemented in Palau to detect cases. In addition to aiding physicians in diagnosing and treating scrub typhus patients more effectively, such surveillance ensures that future outbreaks are detected quickly. Continued surveillance for antibodies to *O. tsutsugamushi* among humans and rodents in various locations throughout Palau will help identify foci of infections and direct aggressive rodent and mite control activities.
